# Family-Centered care in the NICU: an integrative literature review

**DOI:** 10.3389/fped.2026.1826607

**Published:** 2026-05-29

**Authors:** Ronghua Yang, Xia Zhang

**Affiliations:** The First Affiliated Hospital of Hunan Traditional Chinese Medical College (Hunan Provincial Directly Affiliated Hospital of Traditional Chinese Medicine), Zhuzhou, China

**Keywords:** cross-cultural adaptation, family integrated care, family-centered care, neonatal clinical outcomes, neonatal intensive care unit

## Abstract

**Objectives:**

This paper aims to review and summarize the existing research findings on Family-Centered Care (FCC) and Family-Integrated Care (FICare) in Neonatal Intensive Care Units (NICUs), evaluate their impacts on infants and their families, explore the facilitators and barriers during implementation, and discuss the applicability of these care models.

**Design:**

We formulated strict inclusion and exclusion criteria for literature search and screening. Eligible studies were selected and collated in accordance with these criteria. Meanwhile, the measurement tools and research methods applied in the included literature were systematically sorted out, and a complete research framework was established.

**Data sources:**

Literature was initially retrieved through databases and supplemented by snowball sampling. After removing duplicates, the remaining literature was screened by title and abstract, followed by full-text evaluation. A total of 33 methodological studies were finally included for analysis, covering randomized controlled trials, cohort studies, qualitative studies and other research types.

**Review:**

FCC takes the family as the core and builds a collaborative partnership between healthcare providers and families. Family-Integrated Care has formed a more standardized implementation framework. Research evidence shows that FCC and FICare can accelerate infants’ clinical growth rate, shorten hospital length of stay and improve breastfeeding rate, while reducing parental stress and effectively alleviating parental mental health problems. This review further identified the core facilitators for the implementation of these care models and summarized the major implementation barriers. Relevant studies confirm that the models have good applicability in countries such as China and India, although family financial burden affects their long-term implementation.

**Conclusions:**

FCC and FICare can significantly improve the clinical and developmental outcomes of NICU infants, enhance the efficiency of infant recovery and the mental health of parents. The promotion of this model requires strengthened training for medical staff, optimized diagnosis and treatment environments, as well as relevant adjustments based on regional culture and economic levels. Despite certain implementation challenges, they remain high-quality care models that meet the holistic needs of neonates and their families. In the future, digital technology will be used to explore their impact on long-term neurodevelopment of neonates, and a standardized evaluation system will be established to facilitate clinical application.

## 1 Introduction

Family-Centered Care (FCC) and Family-Integrated Care (FICare) are two prominent models in neonatal care, yet they differ in their origins, core principles, and operationalization. FCC emerged in the 1980s and 1990s from broader movements in pediatric and adult healthcare that recognized families as essential partners in care (1, 2). Its core principles include respect for family values and choices, collaboration between healthcare providers and families, information sharing, and the creation of supportive care environments (3, 4). In the NICU context, FCC manifests as open family access, participation in medical rounds, and shared decision-making, but the primary responsibility for clinical care remains with the professional staff (5).

In contrast, FICare is a more recent and structured model, originally developed in Canada around 2010 and subsequently adapted internationally. It is built upon the principles of FCC but extends them by actively integrating parents as primary caregivers for their infants, under the training and supervision of nurses. Key components of FICare include structured parent education, daily skin-to-skin contact, documentation of care activities, and parental participation in medical and nursing handovers. Whereas FCC emphasizes collaboration and partnership, FICare operationalizes family integration through a formal transfer of specific caregiving tasks to parents, positioning them as an integral part of the clinical team. With the continuous advancement of clinical practice, several variant forms of this model have gradually emerged. For instance, such models include mobile-enhanced Family Integrated Care (mFICare), which leverages digital applications to deliver high-quality educational resources and convenient communication channels to parents (6, 7), and Alberta FICare™, a version specifically tailored for level II NICUs (8, 9).

Despite these distinctions, FCC and FICare share similar philosophical foundations—namely, respecting family autonomy, recognizing the parent-infant dyad as the unit of care, and aiming to improve both infant and family outcomes. Both models have been associated with improved weight gain, reduced length of stay, enhanced parental confidence, and decreased parental stress (1, 5). It is worth noting that the core philosophy of FCC has also been fully integrated into developmental care models. This model closely combines neuroprotective environmental adjustments with active family participation, forming an efficient integrated care system (10, 11). However, because FICare involves a more intensive parental role, it may have different facilitators, barriers, and implementation requirements compared to FCC. Given the growing international interest in both models and the lack of a recent synthesis that explicitly compares them across multiple outcomes and contexts, this review aims to synthesize and evaluate the existing evidence on both FCC and FICare, including their impacts, facilitators, barriers, and applicability in contemporary NICU settings.

This study systematically synthesized the core findings 53 relevant studies, focusing specifically on three key dimensions of the research. To start with, This review evaluates how the FCC/FICare model impacts infant health outcomes and the overall well-being of families. Next, An in-depth exploration is carried out of the various factors that facilitate the implementation of this model, as well as the practical barriers that often emerge in the process. Finally, This review examines the adaptability of the FCC/FICare model across different cultural contexts and diverse healthcare systems, along with the corresponding adjustment strategies that can be adopted.

## 2 Methodological overview of the included literature

### 2.1 Search strategy and study selection

To ensure both transparency and systematicity throughout the literature synthesis process, A standardized approach was developed for screening and selecting studies on both Family-Centered Care (FCC) and Family-Integrated Care (FICare) in the neonatal intensive care unit (NICU) setting. The inclusion and exclusion criteria applied to all identified records are summarized in Table 1. These criteria were designed to capture empirical and synthetic evidence addressing either FCC or FICare, or both models, thereby aligning with the primary objective of this review to synthesize existing evidence on both care approaches, evaluate their impacts, and explore facilitators, barriers, and applicability.

**Table 1:** Inclusion and exclusion criteria for studies on FCC and/or FICare in the NICU.

Category	Inclusion Criteria	Exclusion Criteria
Study Design	Randomized Controlled Trials, Cluster Randomized Controlled Trials, Quasi-experimental studies (pre-post, non-randomized controlled), Cohort studies, Case-control studies, Mixed-methods studies, Qualitative studies (interviews, focus groups), Systematic reviews/protocols, Cross-sectional surveys.	Non-research articles (e.g., commentaries, editorials), reports not providing original data or a clear methodology.
Study Population (Infants)	Preterm infants (gestational age ≤37 weeks) or sick newborns admitted to a NICU, particularly Very Low Birth Weight Infants, those with Bronchopulmonary Dysplasia, or other conditions.	Non-NICU populations, infant groups without clear diagnosis or gestational age information.
Study Population (Families)	Parents (including mothers and fathers) or primary caregivers (e.g., grandparents) of infants hospitalized in the NICU. Studies may focus specifically on the experiences and outcomes of fathers or mothers.	Studies not involving family participants or focusing solely on healthcare professional perspectives (unless as a secondary part of an implementation study).
Intervention	Implementation of a FCC, Family Participatory Care, or FICare model. Includes core components such as specific education sessions, psychological support, environmental modifications (e.g., single-family rooms), and technological support (e.g., mobile-enhanced FICare/mFICare).	Studies describing only standard care, lacking a structured FCC/FICare intervention, or with unclear interventions.
Comparator	Standard care, routine care, or FCCand/or FICare (as a less intensive intervention comparator) in the NICU.	Descriptive or qualitative studies without a control group (as per their design).
Outcomes	Reports infant clinical outcomes (e.g., weight gain, length of stay, infection rates, feeding), family psychosocial outcomes (e.g., parental stress, anxiety, depression, self-efficacy, satisfaction), or implementation process outcomes (e.g., feasibility, acceptability, barriers, and facilitators).	Studies not reporting relevant clinical, psychological, or process outcomes.
Publication & Language	Peer-reviewed articles published in English.	Non-English articles, conference abstracts, theses, book chapters, unpublished manuscripts.

The literature search and screening followed the criteria below. Studies were included if they met the following criteria: (a) published between 2005 and 2025; (b) written in English; (c) A systematic literature search was conducted in seven electronic databases: PubMed, MEDLINE (Ovid), CINAHL (EBSCO), the Cochrane Library, Web of Science, Scopus, and Embase. The search strategy combined keywords related to FCC, FICare, neonatal care, family involvement, and parent-infant interaction.

### 2.2 Screening process

**Figure 1:**
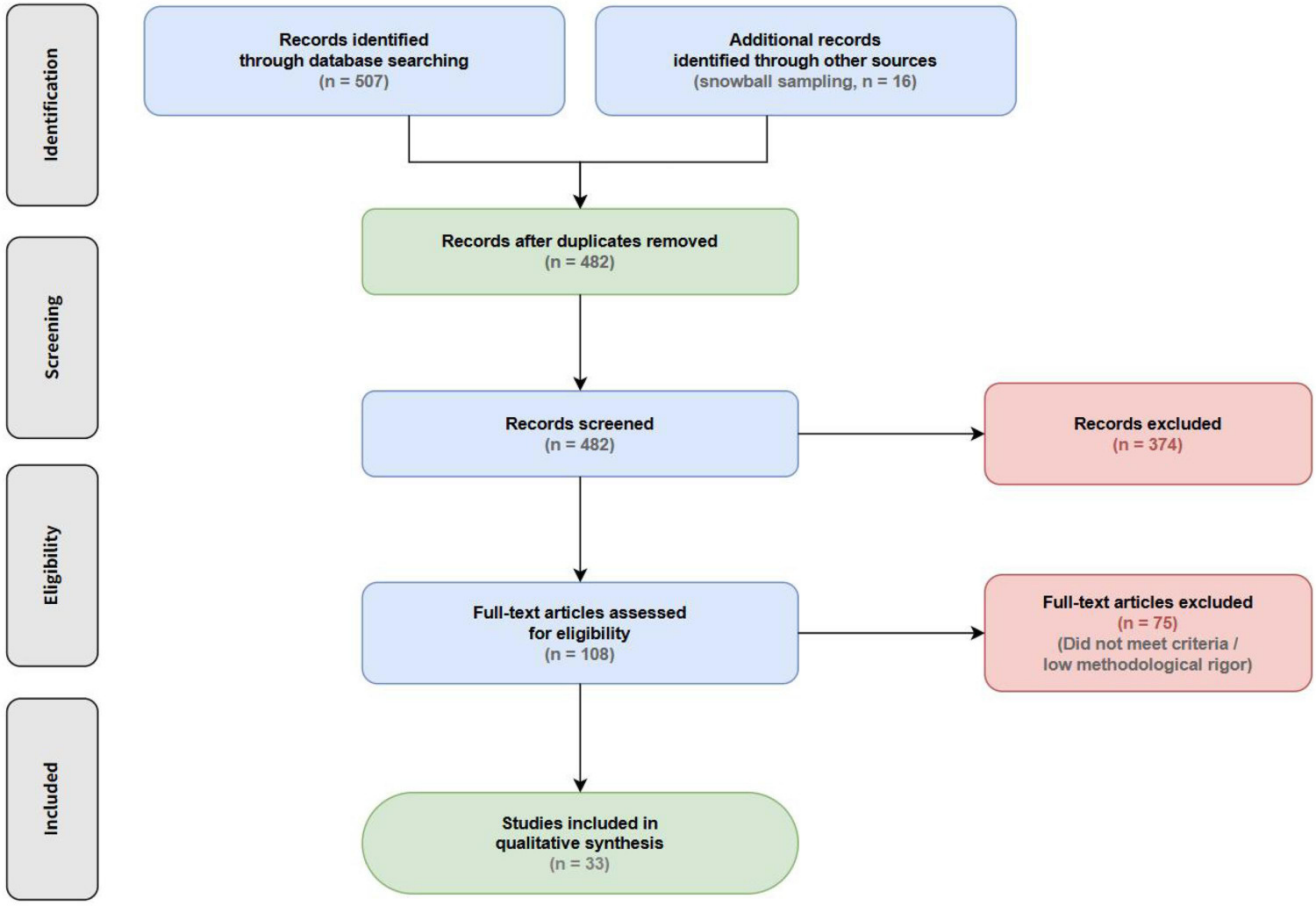
The PRISMA flow chart.

The title/abstract screening and full-text screening were performed by the first author based on the predefined inclusion and exclusion criteria (Table 1). To ensure reliability, all screening decisions were reviewed by the corresponding author. Any disagreement between the two authors was resolved through discussion. The PRISMA flow diagram (Figure 1) summarizes the number of records identified, screened, and included.

### 2.3 Data extraction and thematic analysis

A standardized data extraction form was developed and piloted on three randomly selected studies. Data extraction was performed by the first author and verified by the corresponding author. The extracted data were synthesized using thematic analysis. The process involved: (a) open coding of findings from each study; (b) grouping similar codes into preliminary categories; (c) reviewing and refining categories into final themes through discussion between the two authors. Consistency was ensured by regular discussions and by documenting all coding decisions.

### 2.4 Critical appraisal of included studies

A critical appraisal of the methodological quality of included studies was conducted to assess risk of bias and to inform the strength of the evidence synthesis. The Mixed Methods Appraisal Tool (MMAT) version 2018 was used for empirical studies (12), and the AMSTAR 2 checklist was used for systematic reviews (13). The appraisal was performed by the first author and verified by the corresponding author; any disagreements were resolved through discussion.

### 2.5 Ethical considerations

As this study is a literature review of previously published articles, formal ethical approval was not required.

The PRISMA flow diagram (Figure 1) provides a full and detailed overview of the study selection process for studies on Family-Centered Care (FCC) and/or Family-Integrated Care (FICare) in the NICU (14). Based on the predefined search strategy we developed, along with the use of classification filters, the initial database search yielded 507 articles in total; an extra 16 articles were obtained via snowball sampling. After removal of 41 duplicate records, 482 unique articles were left for further evaluation. An initial screening of titles and abstracts was subsequently carried out of titles and abstracts, excluding those studies that did not meet our eligibility criteria—this resulted in 108 articles moving on to full-text assessment. Finally, after excluding studies that either did not meet the inclusion criteria or lacked methodological rigor, 33 articles were included in the qualitative synthesis.

Table 2 summarizes the quantitative findings, measurement strategies, and qualitative methods adopted in all studies included in this analysis, providing core support for the evidence presented and discussed in subsequent chapters of the paper.

**Table 2:** Quantitative outcomes and measures .

Study Type	Primary Domains/Themes Measured	Specific Measurement Tools/Methods	Representative Citations
Quantitative Studies	**Infant Growth Metrics**	Weight gain velocity, change in weight Z-score, discharge weight, time to regain birth weight.	(15–17, 20, 21, 55)
	**Infant Morbidity & Hospitalization**	Incidence of nosocomial infections, Necrotizing Enterocolitis, Bronchopulmonary Dysplasia, Retinopathy of Prematurity; Total length of hospital stay, duration of oxygen therapy, time to achieve full enteral feeding.	(8, 15, 16, 18–20)
	**Feeding Outcomes**	Exclusive breastfeeding rate, any breastmilk feeding rate, daily milk intake, feeding intolerance rate, breastfeeding self-efficacy.	(10, 15, 16, 18, 22, 23)
	**Parental Psychological Status**	Parental Stress: Parental Stressor Scale: NICU. Anxiety & Depression: Hospital Anxiety and Depression Scale, State-Trait Anxiety Inventory, Patient Health Questionnaire. Self-Efficacy: Perceived Parenting Self-Efficacy Scale, Breastfeeding Self-Efficacy Scale. Family functioning and satisfaction.	(22, 25–29)
	**Parental Engagement & Readiness**	Parent participation (e.g., CO-PARTNER tool), readiness for discharge scores.	(25, 30, 31)
Qualitative Studies	**Parent/Family Experiences & Perceptions**	Semi-structured interviews, focus group discussions exploring NICU experiences, feelings about involvement in care, coping strategies, and perceptions of FCC/FICare. Analytical methods include thematic analysis, content analysis, interpretive description, or grounded theory.	(6, 32, 42, 48–50)
	**Healthcare Professionals’ Perspectives & Experiences**	Semi-structured interviews, focus groups to understand attitudes towards FCC/FICare, barriers and facilitators to implementation, and experiences with role changes. Analysis using theoretical frameworks such as the Consolidated Framework for Implementation Research.	(33, 34, 39, 40, 52)
	**Implementation Process Evaluation**	Qualitative components within mixed-methods designs to assess feasibility, acceptability, fidelity, and to explain quantitative findings.	(22, 34, 43, 54)
Mixed-Methods Studies	**Comprehensive Evaluation**	Combines quantitative measures with qualitative methods for a holistic understanding of intervention effects, mechanisms, and contextual factors.	(22, 32, 43, 53)

It can be clearly seen from the above tables that the existing research findings adopt a diversified range of research methods: they include not only rigorously designed clinical trials but also qualitative studies that deeply explore the relevant interests. Such a diversified and multi-level methodological system enables us to comprehensively and systematically grasp the implementation processes and effects of FCC and FICare, as well as the optimization and adjustment schemes required for the models to adapt to specific application scenarios. Subsequent sections will provide a detailed analysis.

## 3 Results

### 3.1 Impact on infant clinical and developmental outcomes

Numerous studies have clearly demonstrated that both FCC and FICare can significantly optimize key health indicators in neonates. Compared with neonates receiving routine care, those undergoing FCC showed faster weight gain, better overall growth, and a significantly shorter duration of oxygen therapy. Of special interest, data from multiple studies consistently indicate that neonates whose families are actively involved in FCC care have a markedly shorter hospital stay (8, 15–18). Furthermore, FCC can significantly reduce the incidence of nosocomial infections in neonates (15, 19). In addition, this model can effectively lower the risk of common severe diseases in preterm infants (20), including bronchopulmonary dysplasia, necrotizing enterocolitis, and retinopathy of prematurity. For very low birth weight infants, a high-risk population, Family-Integrated Care plays an irreplaceable protective role and can effectively prevent extrauterine growth restriction in these infants at discharge (21). In terms of feeding outcomes, infants receiving FCC show a significantly higher breastfeeding rate at discharge compared with those receiving conventional care (15, 16, 18, 22, 23).

It is important to note that most of the current evidence on FCC originates from tertiary (Level III) NICUs. However, clinical studies in Level II NICUs have also verified the practical value of this model, such as reducing infants’ hospital stay (8). Nevertheless, there are inconsistencies among different studies regarding its effectiveness in promoting early weight gain in infants (24). Additionally, emerging evidence continues to reveal the potential positive impacts of FCC on infants’ neurodevelopment: on one hand, infants receiving FCC care achieve relatively higher scores in mental development index and psychomotor development index (15); on the other hand, when the FCC model is integrated with developmental care principles, infants’ Bayley Scale scores are further improved (11).

### 3.2 Impact on parent and family outcomes

The positive impacts of FCC have permeated deeply into parents’ lives and even the whole family system. When compared with parents who receive conventional standard care, those actively engaging in the FCC model tend to keep lower levels of stress, anxiety, and depression consistently (25–28). This reduction in stress can be partly credited to the FCC model's unique design—it not only reinforces the supportive role of nurses during the care process but also keeps optimizing a variety of family-oriented care practices (29). At the same time, parents’ self-efficacy, professional care capabilities, and confidence in taking care of their infants are notably boosted (16, 25), which in turn enhances their readiness to handle home care after discharge effectively (30). Besides, the FCC model exerts a vital role in fostering the emotional connection between parents and their infants (25).

Within the traditional care setting of NICU, fathers’ needs and mental health have frequently been neglected. However, when fathers are integrated into the care system of the FCC model, their stress levels and depressive symptoms are alleviated—and fathers’ active participation acts as a key mediating factor driving these positive changes (25, 31). Parents participating in FCC gave higher ratings for both overall family conditions and the care services they received (16, 28). Relevant qualitative studies further found that FCC enables mothers to go through the process of “returning home” in a more positive state and better adjust their own recovery rhythm (32). In addition, the positive effects of FCC are not confined to the hospital stay, but can extend beyond the infant's discharge. Research data show that this care model effectively relieves mothers’ post-traumatic stress disorder (PTSD) and depressive symptoms. The psychological improvement is especially significant for mothers who endured severe stress during their stay in the NICU (27).

### 3.3 Facilitators, barriers, and implementation processes in FCC and FICare

Based on the synthesized evidence from the included studies, the following facilitators, barriers, and implementation processes were identified to align with the study objectives.

#### 3.3.1 Facilitators

Key facilitators promoting successful FCC and FICare implementation include: (a) active family involvement, particularly mothers’ engagement in daily care routines and feeding, which directly enhances infant outcomes (15, 16, 18, 22, 23); (b) the supportive role of nurses in reinforcing family-oriented care practices and reducing parental stress (29); (c) integration of developmental care principles with the FCC model, leading to additional neurodevelopmental benefits (11); and (d) structured inclusion of fathers, whose participation alleviates their own stress and depression while mediating positive family dynamics (25, 31).

#### 3.3.2 Barriers

Several barriers to FCC and FICare implementation were identified across studies: (a) most evidence originates from tertiary (Level III) NICUs, with limited validation in Level II settings, suggesting potential resource or training constraints for broader scaling (8); (b) inconsistencies in study findings regarding FCC’s effectiveness in promoting early weight gain, indicating that outcomes may vary by context or patient subgroup (24); and (c) traditional NICU practices that often neglect fathers’ needs, posing a barrier to fully inclusive family-integrated care (25, 31).

#### 3.3.3 Implementation processes

The implementation processes of FCC and FICare, as described in the literature, typically involve: (a) structured integration of families into daily care activities from admission through discharge, including feeding, oxygen therapy management, and infection prevention (15–19); (b) continuous nurse-family collaboration and education to build parental self-efficacy and care competencies (16, 25, 30); (c) incorporation of psychological support mechanisms to address parental stress, anxiety, depression, and post-traumatic stress disorder (27, 32); (d) adaptation of the model to different NICU levels (e.g., Level II) with tailored strategies, such as reducing hospital stay (8); and (e) when combined with developmental care, additional process adjustments like individualized neurodevelopmental support to further improve Bayley Scale scores (11).

## 4 Discussion

### 4.1 Implementation: facilitators, barriers, and process

To successfully implement FCC and FICare in the NICU, in addition to comprehensive and multi-dimensional planning, long-term investment is also needed to transform the inherent work culture within the NICU and help medical staff adapt to the new care model. Studies have confirmed that there are three core factors supporting the effective implementation of this model: first, strong clinical leadership combined with collaborative consensus across multidisciplinary teams; second, systematic training programs tailored to the needs of all NICU staff; and finally, the development of accessible, user-friendly educational resources and standardized care guidelines for parents, which is an indispensable key component (33–35).

In terms of implementation strategies, effective clinical practice measures mainly include establishing sound communication mechanisms, offering parental education courses, and building collaborative systems (8, 36, 37). In addition, the physical environment of the NICU is also an important facilitating factor, among which single-family rooms play a relatively critical role. Such wards not only allow parents to accompany their neonates around the clock but also ensure the privacy of parent-infant interactions (5, 25, 38).

Although Family-Centered Care (FCC) and Family-Integrated Care (FICare) have significant application value, their promotion still faces many obstacles. Among medical staff, their resistance to this model mainly stems from various concerns, including worries about increased workload, reduced professional autonomy, and anxiety over infant safety (33, 39, 40). Meanwhile, inadequate hardware facilities, insufficient human resources, and a lack of dedicated funding are common challenges in most medical institutions (33, 34, 41). Therefore, adequate staffing and appropriate facility conditions are necessary prerequisites for the effective implementation of FCC and encouraging families to actively participate in care (42).

In regions with long-standing strict visitation restrictions, such as China and parts of India, breaking inherent institutional norms and policy frameworks represents a core barrier to model implementation (18, 43, 44). Process evaluation findings show that the effective rollout of FCC largely depends on the organizational readiness of medical institutions, the compatibility of the model with existing hospital workflows, and continuous reflection, evaluation and optimization during implementation (34, 41).

What's more, sustained relevant education, the setup of a supportive leadership system, and a positive organizational atmosphere are central to tackling the above-mentioned issues and sustaining long-term changes in nursing practice (34, 41, 45). Meanwhile, this implementation process also leans on a long-term, multi-faceted evaluation mechanism—one that comprehensively covers outcome indicators related to infants, families, and staff—to guarantee the effectiveness of the FCC model (46).

### 4.2 Cultural, contextual, and economic adaptations

FCC has been adapted and researched under diverse cultural backgrounds and socioeconomic conditions, fully demonstrating its strong flexibility. Looking back at China's practice, historically, parents’ visiting rights in NICUs were extremely restricted. The introduction of the FICare model was inseparable from large-scale staff training and the revision and improvement of relevant policies. Ultimately, this model not only effectively improved infants’ health outcomes but also successfully enabled parents to deeply participate in the infant care process (18, 33, 44).

In India, a culturally adapted FCC program has shown good feasibility and acceptability. Focusing on hand hygiene (as a core measure for infection prevention) and basic care skills, the program not only increased the pre-discharge breastfeeding rate but also did not lead to a rise in the risk of nosocomial infections (23, 43, 47). Relevant studies from Ghana, Iran, and Latin America have pointed out that the implementation of FCC must respect local cultural and religious customs, strive to address the economic difficulties faced by families, and tailor exclusive communication and education plans according to actual circumstances (48–50).

Economic factors are crucial to the sustainable development of the FCC model. Although FCC can help hospitals save costs by shortening infants’ length of stay (8), it cannot exempt families from the high expenses they need to bear themselves, such as transportation, accommodation, and catering costs. For families living far away from hospitals, this economic pressure is even more pronounced (51). Therefore, it is suggested that targeted economic support should be provided to offset these additional expenditures, allowing all families to equally enjoy the benefits brought by the FCC model (51).

### 4.3 Staff perceptions, training, and future directions

The attitudes and preparedness of healthcare professionals are the core determinants of the successful implementation and effectiveness of the FCC model. Targeted educational programs and awareness-raising training can effectively enhance staff's recognition of the model, enrich their professional knowledge reserves, and strengthen their practical skills in supporting families’ participation in care (33, 37, 45). However, while most staff acknowledge the numerous benefits that the FCC model brings to families, some still hold concerns, specifically focusing on issues such as ambiguous role positioning, tight work schedules, and difficulties in properly managing complex family relationship dynamics (33, 39, 40, 52).

Looking ahead, research and innovation in the FCC field can move forward in three key directions. For one direction, we should fully tap into digital technology tools—mobile applications and telehealth services, to name a few—to further boost the effectiveness of parent education, provide families with access to virtual participation in clinical rounds, and keep close family connections intact. Significantly, the research directions related to FCC have demonstrated significant value in practical application during the COVID-19 pandemic (6, 7, 36, 53). There are currently two core research priorities in this field. The first is to strengthen research efforts, specifically including prioritizing follow-up studies on infants’ long-term neurodevelopmental outcomes, conducting rigorous cost-effectiveness analyses, and establishing a Core Outcome Set to unify the evaluation criteria for FCC-related research (54). The second is to deepen specialized research: on the one hand, optimizing FCC care plans for critically ill neonates and infants requiring surgical intervention; on the other hand, developing more inclusive participation strategies to ensure that non-birthing parents and diverse family structures can equally engage in the care process (31).

## 5 Conclusion

A large body of reliable research evidence has demonstrated that FCC and FICare can significantly improve the clinical recovery, developmental progress of ill neonates in the NICU, as well as the psychological status of their parents.

Specifically, the application of these two care models can effectively promote the growth and development of ill neonates, shorten the length of hospital stay, increase breastfeeding rates, while reducing parental psychological pressure and alleviating their negative emotions.

The successful implementation of FCC is not a simple nursing adjustment, but a complex transformation involving nursing philosophy and service models. This process requires efforts in strengthening specialized training for medical staff, optimizing the medical environment, and promoting relevant policy reforms. Meanwhile, localized adjustments of the FCC model should be carried out in combination with the regional culture, social environment and economic development level of different regions, so as to make it better fit the local actual medical needs.

Although existing studies have fully shown that the FCC model faces numerous difficulties and challenges in its promotion and implementation, it is still a high-quality care model that balances humanistic care and medical ethical principles. It can precisely meet the multi-dimensional needs of critically ill neonates and their families in physical health, psychological status and social adaptation, laying a solid foundation for the lifelong healthy development of neonates and family well-being.
